# Multielectrode Arrays as a Means to Study Exocytosis in Human Platelets

**DOI:** 10.3390/bios13010086

**Published:** 2023-01-04

**Authors:** Rosalía González Brito, Pablo Montenegro, Alicia Méndez, Valentina Carabelli, Giulia Tomagra, Ramtin E. Shabgahi, Alberto Pasquarelli, Ricardo Borges

**Affiliations:** 1 Pharmacology Unit, Medical School, Universidad de La Laguna, 38200 La Laguna, Spain; 2 Drug Science Department and NIS Centre, University of Torino, 10125 Torino, Italy; 3 Institute of Electron Devices and Circuits, Ulm University, 89069 Ulm, Germany

**Keywords:** amperometry, chromaffin cells, electrochemistry, exocytosis, secretion, serotonin

## Abstract

Platelets are probably the most accessible human cells to study exocytosis by amperometry. These cell fragments accumulate biological amines, serotonin in particular, using similar if not the same mechanisms as those employed by sympathetic, serotoninergic, and histaminergic neurons. Thus, platelets have been widely recognized as a model system to study certain neurological and psychiatric diseases. Platelets release serotonin by exocytosis, a process that entails the fusion of a secretory vesicle to the plasma membrane and that can be monitored directly by classic single cell amperometry using carbon fiber electrodes. However, this is a tedious technique because any given platelet releases only 4–8 secretory δ-granules. Here, we introduce and validate a diamond-based multielectrode array (MEA) device for the high-throughput study of exocytosis by human platelets. This is probably the first reported study of human tissue using an MEA, demonstrating that they are very interesting laboratory tools to assess alterations to exocytosis in neuropsychiatric diseases. Moreover, these devices constitute a valuable platform for the rapid testing of novel drugs that act on secretory pathways in human tissues.

## 1. Introduction

Exocytosis is the main way in which most neurotransmitters are released, especially biological amines (adrenaline, noradrenaline, dopamine, histamine, and serotonin), the kinetics of which have been mainly characterized by amperometry. However, there are a limited number of human cells that can be used to study exocytosis. Blood platelets, also called thrombocytes, are anucleated cells produced by megakaryocyte budding in the bone marrow. Platelets are irregular structures with a maximal diameter of 2–3 µm. Besides their role in blood hemostasis, platelets are essentially secretory cells with three types of secretory vesicles: α-granules, δ-granules, and lysosomes. Of these, δ-granules accumulate serotonin (5-HT) by taking it up efficiently from blood, such that >99% of the circulating 5-HT is stored in platelets [1]. While any given platelet may contain over 90 α-granules, it will have only 4–8 δ-granules [2], making it difficult to perform biochemical studies on δ-granules, even when using single-cell amperometry. The fusion of δ-granules releases a cocktail of compounds from platelets, including serotonin and histamine, as well as ADP and ATP.

Platelets have been used to model human diseases [3,4,5] and as a means to monitor several functional parameters, such as receptor expression [6]. Indeed, they provide a unique opportunity to explore secretory pathways and exocytosis in human cells that can be readily obtained from blood samples. The behavior of platelets resembles many features of neurons [7], particularly important for the characterization of exocytosis in human diseases and for testing drugs directly on a patient’s own cells. Serotonin is a lipophilic compound that crosses the platelet membrane by passive diffusion or driven by the 5-HT carrier (SERT) [8]. Once internalized, 5-HT is avidly taken up by δ-granules through specific uptake mechanisms, which are probably identical to those used by aminergic neurons or by chromaffin, enterochromaffin, and mast cells [9]. These mechanisms involve membrane carriers, as well as granule core factors that bind to soluble factors and create a dense inner matrix [10]. Most of the carrier proteins present in the granule membrane use the internal H^+^ as a counter-ion. Therefore, granules are acidic and maintain a transmembrane potential (*ψ*) towards the cytosol thanks to a vesicular V-ATPase proton pump that hydrolyzes ATP to pump individual H^+^ ions [11]. Amines are transported into the vesicle by VMAT_2_, exchanging one amine for 2 H^+^ [12]. This exchange system is also followed to accumulate ATP via VNUT [13] and probably to store Ca^2+^ as a result of Ca^2+^/H^+^ exchanger activity [14]. The presence of an inner matrix in secretory vesicles helps drives amine accumulation by forming complexes with soluble species [15].

Most of our understanding of secretory vesicles has come from studies of chromaffin cells, in which secretory vesicles serve to establish an enormous gradient between the granules (≈1 M) [16,17] and the cytosol (≈10–90 µM) [18]. Therefore, any mechanism that is impaired in aminergic neurons and that affects secretory vesicles is likely to be mirrored in platelet δ-granules. Amine secretion, like that of most neurotransmitters, occurs through a calcium-dependent phenomenon referred to as regulated exocytosis. In platelets, it is assumed that the entire 5-HT content is released in an all-or-nothing event [19]. Serotonin is an electroactive molecule, the exocytosis of which can be detected by electrochemical methods [20]. Indeed, amperometry with microelectrodes can detect the release of single exocytotic events with superb sensitivity and temporal resolution [20]. However, single cell amperometry has proven to be inappropriate for routine studies, as it requires expensive equipment and well-trained personnel [21]. In recent years, several multichannel amperometric devices have been developed that enable parallel recording of exocytosis from cell populations [22,23,24], most of which have been tested in chromaffin cells with excellent results [25]. Here we describe the use of a new diamond-based multichannel electrode array (MEA) system to study the functional characteristics of human platelet secretion. This is probably the first time an MEA-diamond based has been used in human cells.

## 2. Materials and Methods

### 2.1. Solutions Used (in mM unless Specified)

Phosphate buffer saline (PBS) – pH 7.4: NaCl (154), KH_2_PO_4_ (1.08), Na_2_HPO_4_ (5).

HEP buffer–pH 7.4 (NaOH): NaCl (140), KCl (2.7), EGTA (5), HEPES (3.8), prostaglandin E_1_ (1 µM), penicillin (100 U/mL), gentamicin (40 µg/mL).

Citrate buffer–pH 7.4 (NaOH): NaCl (150), EDTA (1), Glucose (50), Na^+^-citrate (10), penicillin (100 U/mL), gentamicin (40 µg/mL).

SSP+–pH 7.2 (NaOH): NaCl (69.3), KCl (5), MgCl_2_ (1.5), Na_2_HPO_4_/NaH_2_PO_4_ (28.2), Na^+^-citrate (10.8), Na^+^-acetate (32.5), glucose (5).

SSP++–pH 7.2 (NaOH): NaCl (69.3), KCl (5), MgCl_2_ (1.5), Na_2_HPO_4_/NaH_2_PO_4_ (28.2), Na^+^-citrate (10.8), Na^+^-acetate (32.5), glucose (5), ascorbic acid (10 µM), prostaglandin E_1_ (1 µM), penicillin (100 U/mL), gentamicin (40 µg/mL).

### 2.2. Platelets

Blood samples were obtained from human volunteers and isolated through a variation of the protocol described by Abcam^®^ (Cambridge, UK). Blood was obtained by venipuncture (9 ml in 18.0 mg K_2_-EDTA: BD-Vacutainer^®^, Plymouth, UK) and the tubes were gently rocked for a few seconds before centrifuging for 20 min at 200× *g*. Around two-thirds of the platelet-rich plasma (PRP) supernatant was then transferred carefully to a 15 mL sterile polypropylene tube (JetBiofil^®^, Guangdong, China) using a Pasteur pipette and mixed 1:1 with HEP buffer. After a second centrifugation for 15 min at 100× *g*, the supernatant was again collected in a sterile 15 mL tube and centrifuged for 20 min at 800× *g*. After discarding the supernatant, the pellet was carefully washed twice with 1 mL of citrate buffer, avoiding resuspending the pellet, and the cell pellet was finally resuspended in 5 mL of SSP++ buffer. All centrifugations were performed at room temperature with no brake. The cells were then counted and the concentration measured by turbidimetry (light absorption at 600 nm in a spectrophotometer at a 1:9 dilution with SSP++), adjusting the cell suspension to a final density of 10^5^/µL. Cell suspensions were maintained in a bioreactor tube at room temperature in a humidified incubator, and gently rotated at a 30° angle. Platelets were tested within 24 h of extraction and, in all procedures, care was taken to avoid platelet activation.

All the protocols applied to the human samples were approved by the Ethical Committee of the Canary Islands Health Department and by the Ethical Committees of the University Hospital and the University of La Laguna (Protocols CHUC_2020_80 and CEIBA2020-0430, respectively).

### 2.3. MEA Recording

The procedure to manufacture the 16-electrode boron-doped diamond (BDD) on amorphous quartz MEA and the other details of the three lithographic steps have been described elsewhere [22,24]. When transparency is not required (as here), BDD MEAs can be manufactured on silicon wafers using a simpler approach that requires only two lithographic steps (see Supplementary Materials). For recording, the MEA is connected electrically to a tailored read-out board placed inside a shielding box that acts as a Faraday cage. These front-end electronics contain the potentiostat and the amplifiers for each channel (i.e., 16 low-noise transimpedance amplifiers with an input bias current of ~2 pA and a gain of 1 GΩ), followed by 4th order anti-aliasing Bessel low-pass filters with a cut-off frequency of 6 kHz. The filtered signals are then acquired with an optically isolated 16-bit analog/digital converter (USB6216 multi I/O device: National Instruments, Austin, TX, USA), working over an input range of ±1 V and at a sampling rate of 24 kHz per channel, then digitally low-pass filtering at 1.6 kHz and adapting to an output sampling rate of 4 kHz.

The buffered, optically isolated 16-bit digital/analog converter (included in the same USB multi I/O device) provides the bias voltage for catecholamine oxidation over the common quasi-reference Ag/AgCl electrode immersed into the working solution. Both the data acquisition and the oxidation potential are controlled by software developed in a LabView^®^ environment (National Instruments). Beside the aforementioned optical isolation, both units in the signal chain (i.e., front-end and USB device) are powered electrically by rechargeable batteries to further reduce the risk of picking up external electrical noise and interference.

**Figure 1:**
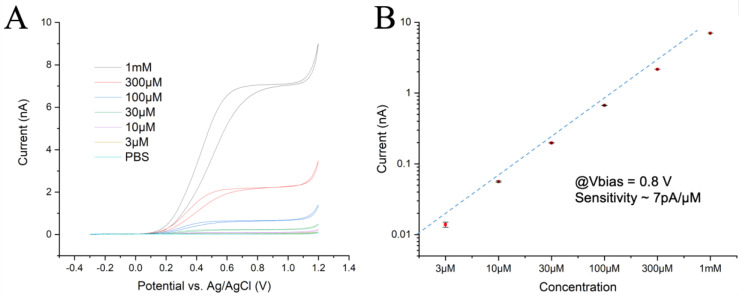
(**A**) CV plots recorded at 50 mV/s with 6 different concentrations of adrenaline. (**B**) Current intensities measured at 0.8 V during the rising ramp, with a linear fit. The electrochemical activity drops slightly at the highest concentration.

Before the experiment, platelets were centrifuged for 20 min at 800× *g* and resuspended at a density of ≈5 × 10^7^/MEA in SSP+ buffer, immediately obtaining the recordings. As silicon devices are opaque, an adequate platelet density and distribution was confirmed by observation on a parallel transparent BDD-on-quartz MEA (see Figure 1S: [26]). An +800 mV potential was applied to the electrodes and an Ag/AgCl pellet acted as a reference electrode. Secretion was elicited by pipetting thrombin at a final concentration of 4 U/mL.

### 2.4. Amperometric Data Analysis

The data was analyzed and graphs produced using IGOR-Pro software (Wavemetrics, Portland, OR, USA). The macros and routines used for automatic data management were modified from our previous versions [27]. The macros were written to extract the following parameters from each spike: **I_max_**, maximum oxidation current expressed in pA; **t_1/2_**, spike full width at half maximum (FWHM) expressed in ms; **Q**, spike net charge expressed in pC; and **m**, ascending slope of spike expressed in pA/ms. These macros, including the modifications to MEA analyses, are freely available upon request. All kinetic parameters were calculated as mean values and the discrimination threshold was fixed at 2.5 standard deviations (SD) of the basal noise of the first derivative for each recording. These usually include spikes with an Imax larger than 1.5–2 pA. All those spikes passed the selection criteria: (1) spikes above the detection threshold, (2) with no overlap, and (3) measured parameters not affected by any artifact during recording.

### 2.5. Electrode Calibration

The assembled device was calibrated by cyclic voltammetry to assess its sensitivity to different concentrations of adrenaline. A software-controlled perfusion system with six channels was used for the different concentrations of adrenaline prepared in PBS (3, 10, 30, 100, 300, and 1000 µM), with one channel used for PBS as a wash solution. As 5-HT binds to the electrode surface, it is not suitable for repetitive application of calibration solutions. The perfusion system was operated by a dedicated function integrated into the LabVIEW program used for data acquisition.

The chip’s perfusion chamber was initially filled with PBS and the lowest adrenaline concentration (3 μM) was then introduced into the chamber for 10 s at a flow rate of 0.2 mL/s. The flow was stopped and the Current/Voltage (CV) response was recorded at a scan rate of 50 mV/s over three full cycles to assess any possible tendency towards fouling. Similarly, the CV measurements were repeated for the other five adrenaline solutions at increasing concentrations. The results obtained for one sample channel and the current differences across channels were less than 10% (Figure 1A), with post-calibration tests showing a reduction in responsiveness of less than 10%. These experiments confirmed the expected microelectrode behavior with radial diffusion of the reagents and products, which resulted in a flat plateau of the oxidation current at overpotentials. They also indicated a dynamic equilibrium between the diffusion rate of reactants in the hemispherical volume above the electrode and oxidation rate at the electrode surface. At a potential larger than 1.1 V, oxygen evolution due to water splitting could be observed.

The oxidation plateau was reached at ~500 mV for low concentrations and at ~700 mV for the 1 mM solution. Both the reaction kinetics and total activity were slightly lower at high concentrations, indicating some hindrance in the exchange of reactants at the electrode surface. However, the identical CV curves for all three cycles suggest no significant fouling was observed, even at the highest concentration. A calibration plot was obtained from the value of the oxidation currents in the CV plots at 0.8 V and achieved a linear fit (Figure 1B), reflecting a sensitivity of ~7 pA/µM with a good linearity over more than 2.5 decades.

### 2.6. Statistics

The data sets are expressed as the means ± SEM. The statistical significance between the groups of experiments was assessed with a non-parametric Mann-Whitney rank sum test or a Student’s *t* test as appropriate, based on the D’Agostino-Pearson normality test. All the data were analyzed using Prism^®^ Software (Graphpad Software, San Diego, CA, USA) and the differences were considered significant at the *p* level indicated.

## 3. Results

**Figure 2:**
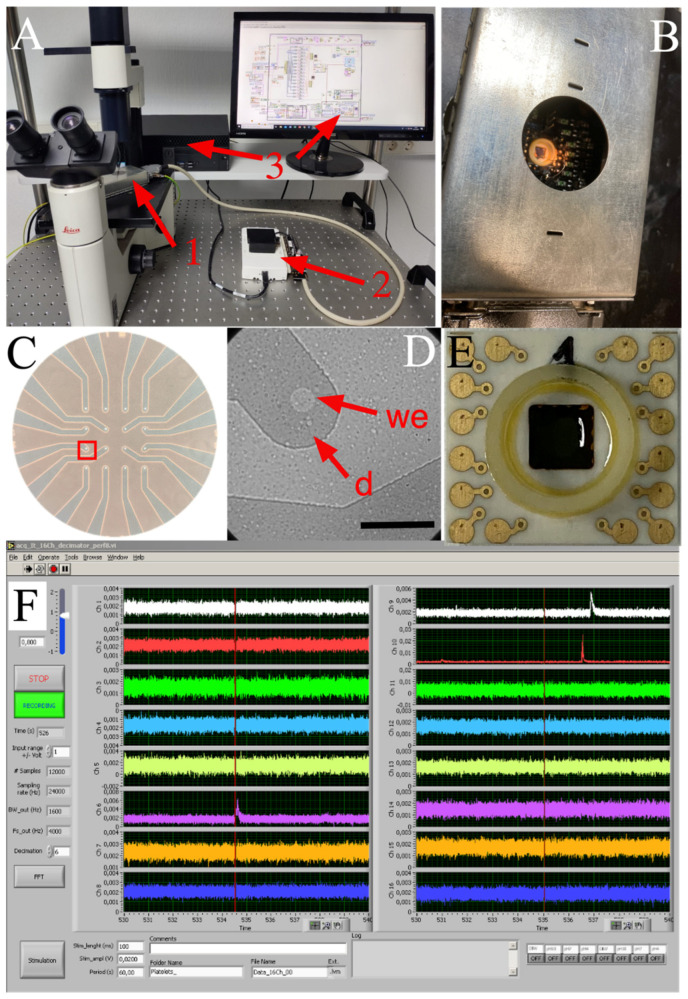
Diamond-based MEA device for amperometric recording of platelet exocytosis. (**A**), General view of the recording system. **1**, When using transparent BDD-on-quartz MEAs, the front-end unit can be placed on the stage of an inverted microscope to examine platelet distribution. **2**, The USB multi I/O unit and the analog low-pass filter plug-in board. **3**, The acquisition program written in LabView^®^ and a PC complete the system. (**B**), Faraday cage containing the MEA chip and amplifiers. (**C**), A BDD-on-quartz MEA showing the 16-electrode layout. (**D**), Magnified view of the red inset in C. Isolated human platelets on a single electrode observed by DIC microscopy: **we**, the open BDD spot acting as a working electrode; **d**, the passivated BDD structure acting as a planar electrical connection. Calibration bar = 50 µm. (**E**), BDD-on-silica MEA showing the gold contact pads. These opaque MEAs have the same 16-electrode layout as in panel C. (**F**), a 10 s recording from the 16 auto-scalable traces show three amperometric spikes in channels 6, 9, and 10.

The MEA system used to record serotonin exocytosis from human platelets is a compact apparatus, consisting of a 16-channel amplifier board situated within a small Faraday box, which is connected to the USB6216 unit and finally to a PC through a USB cable (Figure 2). At the plating density used, several platelets usually attach to the active area of the electrodes without any need for cell positioning. Hence, there is a short preliminary step for the system to settle down, after which a large number of amperometric spikes can be acquired in every recording. The high sensitivity and data throughput make this system suitable for clinical use; moreover, the MEA chips can be rapidly and easily changed in less than 5 minutes.

Under the amplification and bandwidth conditions described above, the typical background noise in the recordings was below 0.9 pA peak-to-peak, corresponding to ~0.15 pA RMS, considerably lower than the RMS noise produced by conventional carbon fiber amperometry (0.4–0.7 pA [28]). In a typical FFT spectra recorded with the system (Supplementary Figure S3), spontaneous spikes were often observed in the absence of stimuli, although secretion was much higher after stimulation with either the calcium ionophore ionomycin (not shown) or the physiological secretagogue thrombin (4 U/mL). The main values of the four major kinetic parameters of secretory spikes validate the results obtained with the MEAs (Table 1). Indeed, our data are in very good agreement with those obtained from human platelets through single-cell recording by conventional glass-encapsulated carbon fiber amperometry and those from other species [19,20,29].

**Figure 3:**
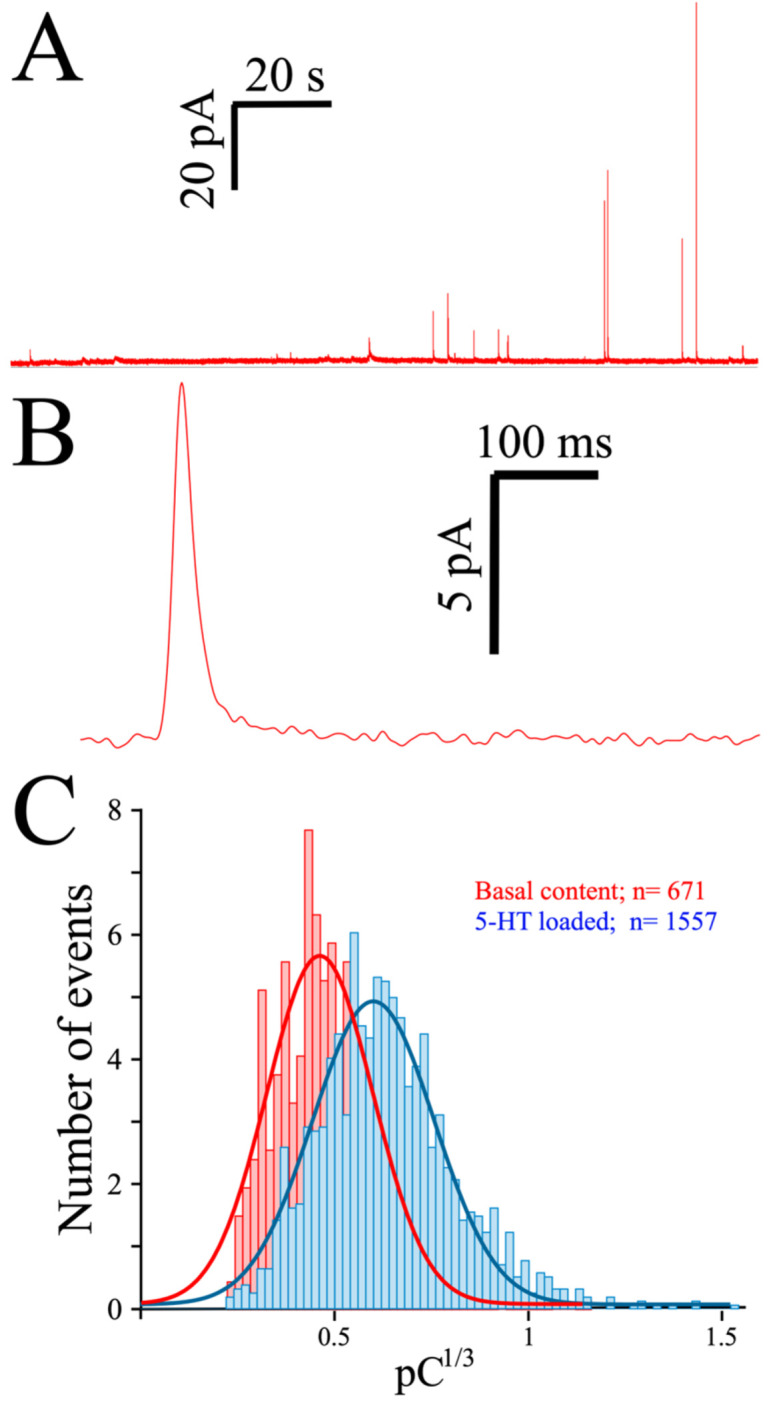
**Exocytosis from human platelets measured with the MEA device**. (**A**), Two-minute recording trace from a single MEA channel of platelets loaded for 2 h with 10 µM serotonin (see Methods). (**B**), A single secretory spike resulting from an exocytotic event. (**C**), Spike charge histograms obtained from control and serotonin-loaded platelets. Superimposed lines show the distribution of the spike charge.

As indicated, spontaneous release often occurs in platelets, as witnessed in a typical MEA recording of 5-HT exocytosis from preloaded platelets (Figure 3A). Contrary to the almost-immediate secretion observed in chromaffin or PC12 cells, the initiation of secretion is usually delayed in platelets by at least 10 seconds when triggered by thrombin, as can be seen in a typical amperometric spike obtained from a preloaded platelet (Figure 3B). To depict the quantal size distribution, we summarize the cubic root of the net charge (**Q**) of the recorded spikes in a histogram (Figure 3C). As only a few spikes are obtained from each platelet, the data was pooled from several consecutive recordings, and the net charge distribution obtained in the absence (red bars) and after exposure to serotonin (2 h, 10 µM: blue bars) is shown.

## 4. Discussion

When secretable species are electroactive, amperometry is currently considered to be the most appropriate technique to analyze exocytosis directly, an approach that combines good sensitivity with superb time resolution. Platelets release serotonin by exocytosis and in some species they also release histamine, making them good candidates for amperometric recordings. Moreover, platelets are the easiest native human cell to obtain for studying exocytosis, as they can be easily isolated from just a few milliliters of peripheral blood. However, human platelets pose several limitations on classical amperometry using carbon fiber electrodes, requiring platelets to be tediously tested one-by-one to record sufficient spikes. This drawback probably explains why only a few research groups currently use platelets to study exocytosis [29]. The main limitations of conventional amperometry are: (i) the small size of platelets, smaller than standard carbon fiber electrodes; (ii) the limited number of secretory spikes that can be recorded during single cell experiments, since a given platelet contains 4–8 δ-granules; (iii) the unwanted activation of adjacent cells by the secretagogue, thrombin, or ionomycin; (iv) unloaded platelets’ production of small secretory spikes that are related to electrical noise, leading most researchers to work with serotonin-preloaded platelets [22].

The recent availability of MEAs capable of performing parallel amperometric recordings has changed this situation, as MEAs can be used for simultaneous recording of exocytosis from several platelets. Moreover, our MEA devices have little electrical noise (Figure S3), which is especially important when studying amperometric spikes as small as those produced by platelets. The low noise observed in our recordings was achieved firstly through the excellent electrical properties of the BDD microelectrodes in conjunction with other novelties, including the short connection paths between electrodes and amplifiers, the optimized low-noise electronics, the use of a battery power-supply, and the use of a compact shielding box acting as a Faraday cage.

Recording from platelets using MEAs proves to be very simple, a procedure that only requires pipetting the platelet solution into the chamber, waiting a few minutes for it to settle down, adding the secretagogue, and recording the exocytotic events. Thanks to this simplicity, the system does not require highly trained personnel nor any of the expensive equipment used in conventional amperometry (electrode puller, grinder, large Faraday cage, anti-vibration table, potentiostat, microscope, micromanipulators, etc.). In addition, this low-cost MEA system is very compact, requiring little bench space; it is very portable and, in combination with a laptop PC, it permits field diagnosis, making related medical tests available even in developing countries.

Parallel recordings allow sufficiently large numbers of amperometric events to be obtained from a platelet suspension in a single run. This is of particular interest for human platelets, as all measurements must be obtained within 48 h of blood donation and the use of MEAs has enabled acceleration of the procedure. As more than one platelet can drop onto the active surface of a given electrode, we successfully recorded amperometric signals from 8–14 of a 16-channel device at appropriate dilutions, yielding 85–180 spikes/assay. In our conditions, a given recording produces enough statistically significant exocytotic data, which means that 1 or 2 MEAs should provide enough information from either a healthy individual or patient.

The kinetic parameters obtained with MEAs are like those acquired with conventional amperometry, though amperometry recordings must be collected by an experienced researcher over several days, while the same quantity of data can be produced in less than 2 h using a diamond MEA. The low-noise recordings of MEA also reduce the time required for data analysis, as spikes are easier to identify from the background. Furthermore, with MEAs we can now measure spikes >1.5 pA as opposed to those of ≈4 pA normally observed by conventional amperometry.

In summary, the MEA system described here provides a novel approach to studying exocytosis in human cells. Given the common features of platelets and other aminergic cells, especially neurons, this offers a unique platform from which to study the basic mechanisms involved in the accumulation and secretion of neurotransmitters in human cells and to test drugs directly on cells from patients.

## Figures and Tables

**Table 1:** Kinetic parameters of amperometric spikes obtained with diamond MEAs. Data from 10 human subjects. Calculations were performed on the N basis (average was found by taking all spikes from each subject and the results were used for statistics). We used the same spikes shown in Figure 3C.; Means ± SEM. *** *p* < 0.005: n.s: not significant. Student’s *t* test.

MEA	Imax (pA)	Q (pC)	t_1/2_ (ms)	m (nA/s)
**Basal**	5.65 ± 0.40	0.16 ± 0.02	19.1 ± 1.46	1.25 ± 0.29
**5-HT loaded**	8.42 ± 0.32 ***	0.33 ± 0.06 ***	31.3 ± 2.0 ***	1.55 ± 0.12 ^n.s^

## Data Availability

The data presented in this study are available in Supplementary Materials.

## Supplementary Materials

The following supporting information can be downloaded at https://www.mdpi.com/article/10.3390/bios13010086/s1, Figure S1—Layout of the MEA: (Left) the chip has a size of 6 × 6 mm^2^. (Right) magnification of the core region showing the 20 µm openings of the microelectrodes, arrayed on a square grid with 200 µm pitch; Figure S2—Schematic representation of the assembled MEA; Figure S3—FFT-plot showing the voltage noise spectrum of all 16 channels of the MEA. The spread of performances among the electrodes is very small. The cursor shows the average maximum noise density of ~4.7 µV/sqrt (Hz); Figure S4—High-speed recording of an exocytotic event. The rise time is an indicator of the dynamic performances of the diamond MEA.
