# Supplementary material for: Multielectrode Arrays as a Means to Study Exocytosis in Human Platelets
Source: Biosensors (Basel). 2023 Jan 4;13(1):86. doi: 10.3390/bios13010086 (PMC9855894; doi:10.3390/bios13010086)
Supplement: Supplementary file 1 [file biosensors-13-00086-s001.zip › biosensors-2072718-supplementary.pdf]

biosensors

Supplementary Materials

Multielectrode Arrays as a Means to Study Exocytosis in Human Platelets

MEA technology

The presented device was realized with only two lithographic masks, four growth steps, and two dry-etching processes. All liquid chemical are from Merk (Darmstadt, Germany), all gases from MTI (Ulm, Germany), boron filaments from Goodfellow (Hamburg, Germany) This fabrication procedure can be described as follows:

1) Cleaning of a 300 µm thick, double-polished 2” wide silicon wafer by sonication in acetone, sonication in isopropanol, removal of contaminations in Piranha solution (3:1 H_2SO_4 + H_2O_2,) and finally oxide removal in hydrofluoric acid

2) Grow intrinsic NCD on the cleaned wafer using a microwave chemical vapour deposition (MWCVD) reactor. First run a 30 min long bias enhanced nucleation (BEN) step in an atmosphere of 0.75 % CH_4 in H_2 at a temperature of 800 °C and pressure of 1.5 kPa, which is then seamlessly followed by a 20 h long step for growing an 8 µm layer of intrinsic NCD, with 1.3 % CH_4 in H_2, at 800 °C and 2.5 kPa.

3) Grow a 300 nm thick hick boron-doped NCD layer (BDD-layer) in a second MWCVD reactor, using solid boron filaments of 200 µm in diameter as dopant source.

4) Perform optical lithography and dry etching for transferring the layout (Fig. S1) of the 16-channel MEAs onto the wafer. 37 devices fit on one 2” wafer. The etch-process (reactive ion etching in O_2/Ar atmosphere) removes completely the unprotected areas of the BDD layer, until reaching the intrinsic NCD. Thus, 16 independent BDD structures remain on the wafer after etching. These 16 structures are radially oriented from the borders to the central area, ending with the tips ordered in a 4x4 matrix with a grid size of 200 µm, as shown in figure 2.

5) Treat the diamond device first in chromo-sulphuric acid and then in “piranha”, to remove the unavoidable graphitic phase from the sample surface and turn the pristine H-termination into O-termination, thus obtaining a clean and hydrophilic surface. This wet chemical process leaves a mix of OH- and O= terminations of the diamond surface, which has proven to be not only very cell-friendly, but also electrochemically stable both in terms of activity and surface potential.

6) Grow a 1 µm thick silicon-nitride passivation layer and then with a second lithographic process define the exposed diamond areas, *i.e.*, the 20 µm wide openings for the electrodes and the contact spots for bonding the chip.

7) Bond the MEA to the carrier board by flip-chip technique, using a silver epoxy glue (EC201 Polytec PT, Waldbronn, Germany) as boding material, attach a glass ring and seal all gaps with non-conducting epoxy, for providing a convenient 200 µL

compartment for electrolytes and cell suspensions. The assembled MEA is schematically represented in Fig. S2.

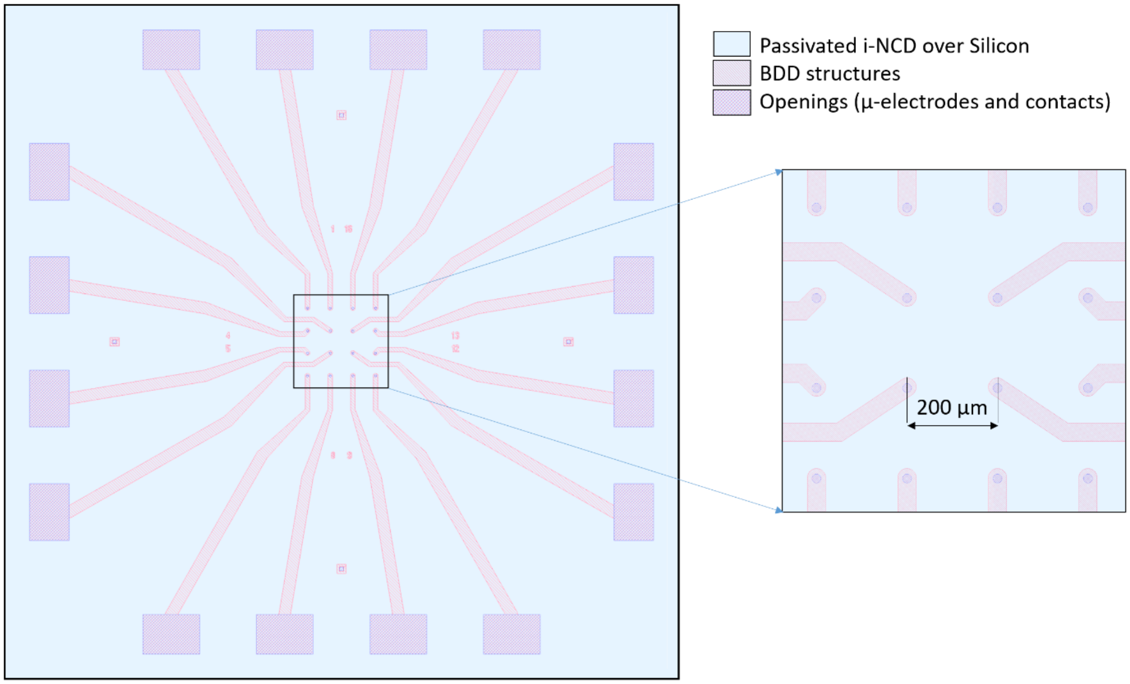

Figure 1. SM – Layout of the MEA: Left) the chip has a size of 6 x 6 mm². **Right)** magnification of the core region showing the 20 μm openings of the microelectrodes, arrayed on a square grid with 200 μm pitch.

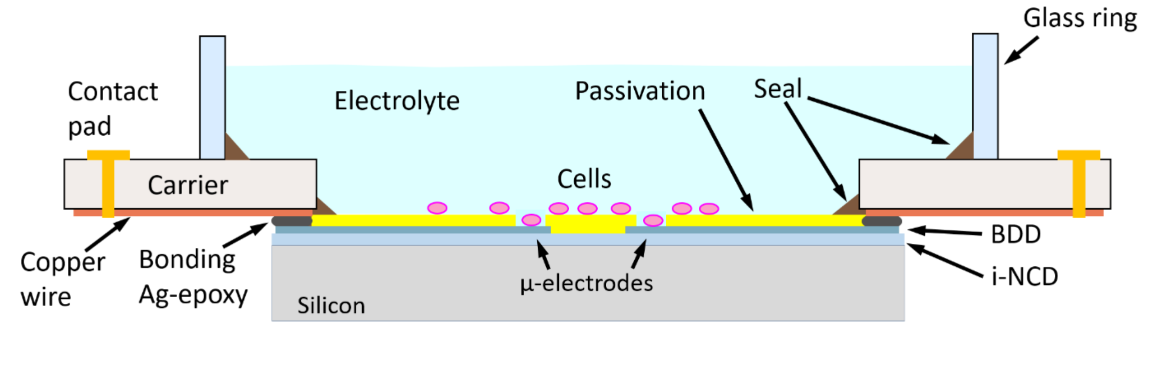

Figure 2. SM – Schematic representation of the assembled MEA.

Background noise

The background noise of the MEA was measured by filling the chamber with PBS and biasing the electrodes at 800 mV.

The results are shown in Fig. S3. The frequency scale is linear because the spectral noise density is defined over a bandwidth of 1 Hz at any frequency. By multiplying the voltage noise density times the square root of the experimental bandwidth of 1 kHz and then dividing it by the value of the feedback resistor (1 GΩ) we obtain the total current noise of ~0.15 pA rms equivalent to ~0.9

pA peak-to-peak.

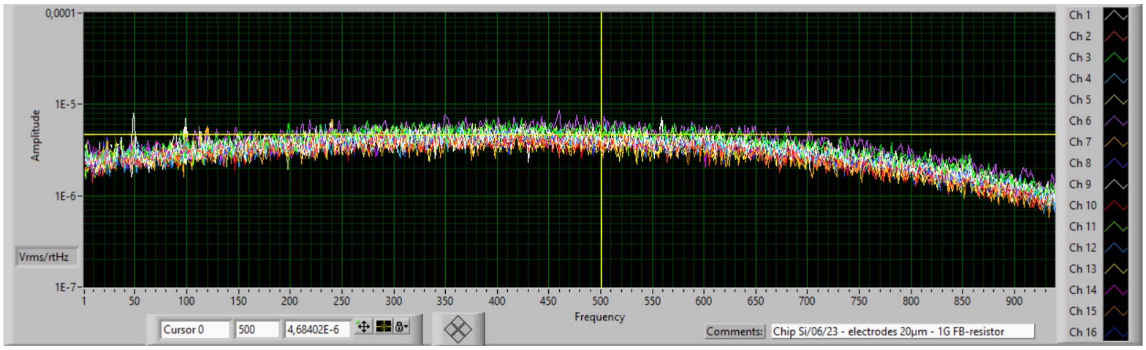

Figure 3. SM – FFT-plot showing the voltage noise spectrum of all 16 channels of the MEA. The spread of performances among the electrodes is very small. The cursor shows the average maximum noise density of ~4.7 µV/sqrt (Hz).

Time response

The dynamic behaviour of the MEA was investigated by performing measurements at the maximum possible sampling rate of 25 kHz per channel and low pass filtering at a bandwidth of 10 kHz, without any further digital filtering and data decimation. Figure 4SM shows the raw data of an exocytotic event recorded under the above conditions. The rise-time, defined as the time between 10% and 90% of the maximum amplitude is ~0.25 ms. Of course such fast events require not only a fast response of the MEA and the electronic chain, but also an optimal location of the cell and the site of the vesicle fusion for minimizing the length of the diffusion path of the released molecules towards the electrode active surface.

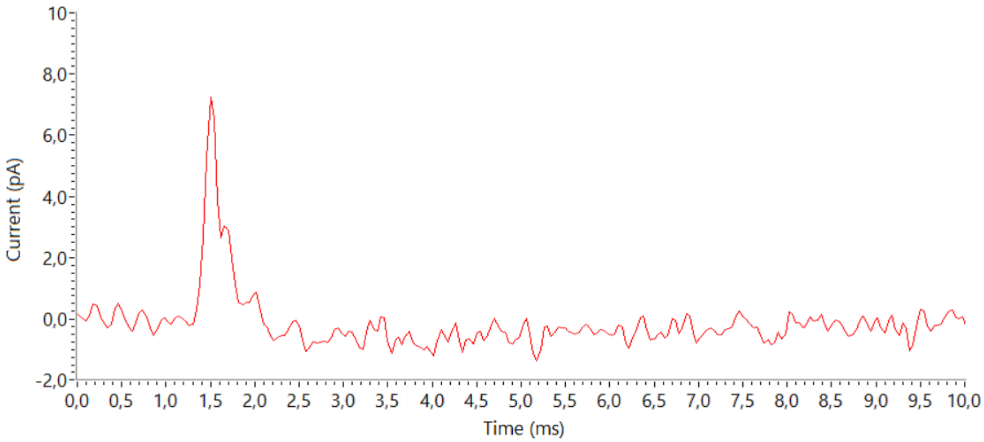

Figure 4. SM – High-speed recording of an exocytotic event. The rise time is an indicator of the dynamic performances of the diamond MEA.
